# Gallic Acid Induces Necroptosis via TNF–α Signaling Pathway in Activated Hepatic Stellate Cells

**DOI:** 10.1371/journal.pone.0120713

**Published:** 2015-03-27

**Authors:** Ya Ju Chang, Shih Lan Hsu, Yi Ting Liu, Yu Hsuan Lin, Ming Hui Lin, Shu Jung Huang, Ja-an Annie Ho, Li-Chen Wu

**Affiliations:** 1 Department of Biochemical Science and Technology, National Taiwan University, Taipei, Taiwan; 2 Department of Education and Research, Taichung Veterans General Hospital, Taichung, Taiwan; 3 Department of Applied Chemistry, National Chi Nan University, Puli, Taiwan; Toho University School of Medicine, JAPAN

## Abstract

Gallic acid (3, 4, 5-trihydroxybenzoic acid, GA), a natural phenolic acid widely found in gallnuts, tea leaves and various fruits, possesses several bioactivities against inflammation, oxidation, and carcinogenicity. The beneficial effect of GA on the reduction of animal hepatofibrosis has been indicated due to its antioxidative property. However, the cytotoxicity of GA autoxidation causing cell death has also been reported. Herein, we postulated that GA might target activated hepatic stellate cells (aHSCs), the cell type responsible for hepatofibrosis, to mitigate the process of fibrosis. The molecular cytotoxic mechanisms that GA exerted on aHSCs were then analyzed. The results indicated that GA elicited aHSC programmed cell death through TNF–α–mediated necroptosis. GA induced significant oxidative stress through the suppression of catalase activity and the depletion of glutathione (GSH). Elevated oxidative stress triggered the production of TNF–α facilitating the undergoing of necroptosis through the up-regulation of key necroptotic regulatory proteins TRADD and receptor-interacting protein 3 (RIP3), and the inactivation of caspase–8. Calmodulin and calpain–1 activation were engaged, which promoted subsequent lysosomal membrane permeabilization (LMP). The TNF–α antagonist (SPD–304) and the RIP1 inhibitor (necrostatin–1, Nec–1) confirmed GA-induced TNFR1–mediated necroptosis. The inhibition of RIP1 by Nec–1 diverted the cell death from necroptosis to apoptosis, as the activation of caspase 3 and the increase of cytochrome c. Collectively, this is the first report indicating that GA induces TNF signaling–triggered necroptosis in aHSCs, which may offer an alternative strategy for the amelioration of liver fibrosis.

## Introduction

Gallic acid (3,4,5-trihydroxy benzoic acid, GA), a natural antioxidant, reportedly undergoes a two-step, one-electron transfer autoxidation to generate GA radicals [1]. The oxidation of GA reportedly initiates at the para-hydroxyl site of a benzene ring to generate semiquinone free radicals, followed by the generation of dehydro-propyl gallate and quinine [2]. Reactive oxygen species (ROS), such as •O_2_^−^, •OH and H_2_O_2_ are concomitantly produced and result in oxidative stress, which can induce cytotoxic activity [3–6]. Cell death caused by GA, such as apoptosis, has been addressed in several cell types, including melanoma, renal, and oral squamous carcinoma cell lines, vascular smooth muscle cells, lung fibroblasts, and leukemia cells [3,7–13]. It is likely that oxidative stress induced by GA autoxidation is a key factor that can cause cell death [6]. However, GA has also been reported to ameliorate hepatic disorders through its antioxidative activity and hepatoprotective effects [14]. Thus, GA may critically maneuver its anti—and pro–oxidative capacity in hepatic malfunctions. Additionally, promoted cell death and the inactivation of activated hepatic stellate cells (aHSCs) during the resolution of acute or chronic injury partially explain the attenuation of fibrogenesis [15]. Accordingly, we postulated that GA could regulate aHSCs to attenuate hepatofibrosis through its anti—and pro–oxidative effects.

Hepatic stellate cells—previously called Ito cells, fat-storing cells or lipocytes—play a central role in hepatic fibrosis [16]. HSCs situate at the space of Disse (or perisinusoidal space) between hepatocytes and sinusoids. HSCs mainly function as storage for vitamin A, a mediator of portal venous pressure and hepatic blood flow, and a regulator of retinoid–related homeostasis in their quiescent state. The activation of HSCs caused by oxidative stress may result in hepatic fibrosis, which can be essentially divided into three stages [17]: the initiation, perpetuation, and resolution. Factors such as tumor necrosis factor-alpha (TNF–α), interleukins, fibronectin, transforming growth factor (TGF–β1), and platelet-derived growth factor (PDGF) secreted by Kupffer cells, hepatocytes, leukocytes, and sinusoidal endothelial cells initiate stellate cell activation [18,19]. Autocrine and paracrine loops subsequently serve to perpetuate activation in the aspects of proliferation, chemotaxis, fibrogenesis, contractility, matrix degradation, and retinol degradation. Once activated, aHSCs perform phenotypic and morphologic changes (myofibroblast–like) and are susceptible to proliferative mediators and inflammatory cytokines [17]. In addition, these cells no longer store retinoid; instead, they produce type I collagen, matrix metalloproteinase (MMP; for extracellular matrix degradation), α-smooth muscle actin (for facilitation of migration), and cytokines to promote fibrogenesis [16,19].

It is noteworthy that the antioxidative system of HSCs during activation varies significantly. Catalase, an antioxidant that catalyzes the degradation of extra/intra-cellular hydrogen peroxide and plays a key role in protecting cells against ROS, shows a restrained expression profile in hepatocellular carcinoma [20] and probably in hepatic stellate cell transformation partially due to the increase of catalase gene methylation [21] and the binding of negative regulators to the silencer elements [22,23]. Accordingly, accumulated oxidative stress resulting from GA autoxidation could lead to cytotoxicity, intracellular Ca^2+^ elevation and oxidative stress-induced apoptosis [24].

Recently, a type of programmed cell death, necroptosis, has been identified [25,26]. Necroptosis, similar to apoptosis, involves defined cell signaling that leads to cell death upon stimulation by ligands such as TNF–α, FasL, and TRAIL. The kinase activities of receptor-interacting protein kinase 1 (RIP1) and RIP3 play central roles in TNF–α–induced programmed necrosis. The signaling mechanisms induced by TNF–α mediate the activation of NFκB and apoptosis through the formation of two protein complexes, I and IIa, respectively, under apoptosis-competent condition. After TNF–α interacting with the TNF receptor (TNFR1), the active TNF–α trimer complex initiates the recruitment of protein molecules such as tumor necrosis factor receptor type 1-associated death domain (TRADD), RIP1, CYLD and several other elements to assemble a complex (complex I). Once dissociated from TNFR1, RIP1 constitutes complex IIa with Fas-associated death domain (FADD) and caspase–8 for the processing of apoptosis. However, under apoptosis-deficient conditions, RIP1 may interact with RIP3 through the RIP homotypic interaction motif (RHIM) domains to form a functional amyloid signaling complex IIb (necrosome) to undergo necroptosis. Additionally, the induction of ROS and calcium-induced lysosomal membrane permeabilization (LMP) through calpain activation has been suggested to critically participate in the execution of programmed necrosis to disrupt cell integrity [26,27].

Herein, we investigated the cytotoxic effect of GA on aHSCs and the underlying molecular mechanisms. Results indicated that GA induced oxidative stress in aHSCs through the inhibition of catalase activity and the promotion of intracellular ROS, lipid peroxides, and oxidative DNA levels. These stresses in turn upregulated the expression of TNF–α, reduced the content of intracellular GSH, and blocked the activation of caspase-8 leading to the initiation of necroptosis characterized by the upregulation of TRADD and RIP3, and the engagement of lysosomal membrane permeabilization modulated by elevated intracellular calcium levels and the activation of calmodulin and calpain 1. In addition, GA-induced necroptosis can be diverted to apoptosis by inhibition of RIP1 activity. These results may offer an alternative strategy for the amelioration of hepatic fibrosis.

## Materials and Methods

### Primary hepatic and hepatic stellate cells isolation and culture

Primary hepatic cells (HCs) and hepatic stellate cells (HSCs) were prepared from Sprague Dawley rat liver as described by Seglen [28] and Kawada et al. [29], respectively. This study was carried out in strict accordance with the recommendations in the Guide for the Care and Use of Laboratory Animals of Taiwan Animal Protect Act. The protocol was approved by the Committee on the Ethics of Animal Experiments of National Chi Nan University (Permit Number: 950102). Carbon dioxide was used for euthanasia, and all efforts were made to minimize suffering. Briefly, the liver was perfused, digested with pronase and collagenase, for 15 min at 37°C at a flow rate of 20 ml/min. and then excised. After further digestion with pronase and collagenase, the resulting suspension was filtered and centrifuged 460×*g* on a 11% (v/v) Nycodenz cushion (Sigma, St. Louis, MO, USA) to isolate HSCs in the upper whitish stellate cell-enriched layer. Resuspended pellets were cultured in DMEM supplemented with 10% FBS and antibiotics (70 mg L^-1^ penicillin and 100 mg L^-1^ streptomycin) at 37°C with a humidified atmosphere of 5% CO_2_. HSCs were activated for 6 passages and used throughout the study. For hepatic cells, after perfusion with collagenase, the liver was excised and dispersed cells in L-15/BSA, followed by sedimentation at unit gravity for 20 min. The supernatant cell suspension was filtered through gauze. The filtrate was washed twice with HBS by centrifugation at 50×*g* for 45 s to remove debris, damaged cells, and non-parenchymal cells. Before seeding, cells were washed once with the culture medium. The range of cell yields was from 4×10^8^ to 6×10^8^ with a survival rate of approximately 95%.

### Cell viability assay

Cell viability was determined by MTT (3-(4,5-Dimethylthiazol-2-yl)-2,5- Diphenyltetrazolium Bromide) assay. Briefly, activated HSCs (aHSCs) were initially plated at a density of 1×10^4^ cells per well in 96-well plates for 24 hrs. The cells were then incubated with designated concentrations of GA and GA analogous (**S1 Fig.**) for 24 hrs at 37°C. MTT (10 μL, 0.5 g/L) solution was then added to each culture well and incubated for another 4 hrs at 37°C. The MTT-formazan crystals produced by viable cells were dissolved by DMSO. The absorbance at 570 nm was monitored with a microplate reader (Bio-Rad, CA, USA). All experiments were performed in triplicate, and the results of treated cells were shown in percentage of untreated control cells.

### Cell proliferation assay

Cells cultured in serum-free DMEM were plated into 96-multiwell plates (5000 cells/well). After incubation for 24 hrs, cells were treated with GA of 0, 25, 50 75 μM. Cell proliferation was measured by using the BrdU cell proliferation assay kit as manufacturer’s instructions (Cell Signaling Technology, Denvers MA). The incorporation of the pyrimidine analogue 5-bromo-2’-deoxyuridine during DNA synthesis in proliferating cells was monitored at 370 nm. All experiments were performed in triplicate, and the results of treated cells were shown in percentage of untreated control cells.

### Cell cycle analysis

At the end of incubation (24 hrs) with GA, the activated HSCs were washed twice with PBS, collected with 0.25% trypsin-EDTA, fixed with ice-cold alcohol (1 mL 75% (v/v)) for 12 hrs at -20°C, and then centrifuged at 380×*g* for 5 min at room temperature. Cell pellets were treated with l mL of cold staining solution containing 20 μg/mL of propidium iodide (PI) and 20 μg/mL of RNase A, and incubated for 15 min in darkness at room temperature. The samples were analyzed by FACSCalibur system (BD Biosciences, Franklin Lakes, NJ, USA) using CellQuest software. Data are representative of at least three independent experiments.

### Lactate dehydrogenase (LDH) release assay

The activity of lactate dehydrogenase (LDH) was measured colorimetrically by LDH assay kit as manufacturer’s instructions (Abcam, Cambridge, UK). Briefly, aHSCs were incubated with designated concentrations of GA for 24 hrs at 37°C. The activity of LDH in culture medium was measured spectrophotometrically recording the rate of change in NADH concentration at a wavelength of 450 nm after interaction with a dye. A NADH calibration curve was constructed to determine LDH activity. One unit of LDH refers to the catalyzation of the conversion of lactate to pyruvate to generate 1.0 μmol NADH per min at 37°C.

### Analysis of reactive oxygen species (ROS) and hydrogen peroxide

ROS was determined using a commercial DCFDA-cellular ROS detection assay kit as manufacturer’s instructions (Abcam, Cambridge, UK). Briefly, aHSCs were plated on a 96-well plate (2.5×10^4^ cells/well). After overnight attachment, cells were treated with GA at designated concentration for 6 hrs, followed by staining with cell permeant reagent, 2’,7’–dichlorofluorescein diacetate (DCFDA) for 45 min at 37°C. Deacetylated DCFDA were fluorescently determined after oxidized by ROS to form 2’, 7’–dichlorofluorescin (DCF) with Ex 495 nm /Em 529 nm. Culture medium and intracellular hydrogen peroxide (H_2_O_2_) of aHSCs were analyzed by using a fluorometric hydrogen peroxide kit as manufacturer’s instructions (Cayman, Ann Arbor, MI). The assay is based on the conversion of 10-Acetyl-3,7-dihydroxyphenoxazine (ADHP) to highly fluorescent resorufin in the presence of horseradish peroxidase (HRP) and H_2_O_2_. The fluorescence of resorufin was read at Ex 530 nm/Em 590 nm. All experiments were performed in triplicate, and the results of treated cells were shown in percentage of untreated control cells after background subtraction.

### Detection of DNA oxidative damage

The detection of DNA oxidative damage was determined by the DNA Damage EIA kit (Cayman, USA) using Anti-8-OHdG monoclonal antibody to competitively bind 8-OHdG. The immune complexes (anti-8-OHdG and free 8-OHdG) were washed away, while antibodies that caught by immobilized 8-OHdG were detected by a horseradish peroxidase (HRP) conjugated secondary antibody, and the absorbance was measured at 415 nm.

### Detection of intracellular glutathione

The concentration of glutathione and oxidized glutathione (GSH/GSSG) was determined by glutathione assay kit as manufacturer’s instructions (Cayman, Ann Arbor, MI, USA). The cell pellet is homogenized in cold phosphate buffer (50 mM, pH 6–7, 1 mM EDTA). The supernatant of the homogenates (10000×g for 15 min) was used to determine GSH/GSSG by an enzymatic recycling method. The protein concentration was determined by the Bradford method.

### Analyses of lipid peroxidation

Intracellular lipid peroxidation of aHSCs was fluorescently (ex. 515 nm; em. 553 nm) measured by the determination of the MDA–TBA complex with fluorometeror (Thermo Scientific) using HPLC with LiChrospher column (RP-18, 5μm, Merck), mobile phase of 25 mM Na_2_HPO_4_–methanol (58/42, v/v) at a flow rate of 1 ml/min. The complex of MDA–TBA was eluted in 4.8 min. A MDA–TBA complex standard curve was constructed for calibration. Additionally, the lipid peroxidation (LPO) assays were performed using a Lipid Hydroperoxide Assay kit (Cayman Chemical). Lipid hydroperoxides were extracted into chloroform and measured by the redox reactions with ferrous ions. Chromogenic reaction was performed at room temperature for 5 min, followed by reading the mixture at 500 nm. The calibration curve was constructed using 13-Hydroperoxy-octadecadienoic acid. Lipid hydroperoxide was expressed as nmol/mg protein.

### Catalase assay

The activity of catalase (CAT) was determined by catalase assay kit as manufacturer’s instructions (Cayman, Ann Arbor, MI, USA). The peroxidatic function of CAT was used for activity evaluation. Cell lysates were incubated with assay buffer, methanol, and H_2_O_2_ for 20 min at room temperature in dark. Potassium hydroxide was utilized to terminate the reaction, followed by the addition of 4-amino-3-hydrazino-5-mercapto-1,2,4-trizazole (Purpald) as chromogen to interact with the produced formaldehyde for 10 min. The reaction mixture was measured spectrophotometrically at 540 nm. CAT activity was expressed as nmol/min/mL. All experiments were performed in triplicate, and the results of treated cells were shown in percentage of untreated control cells.

### Catalase transfection

Transfection was performed using Lipofectamine 2000 (Invitrogen, Carlsbad, CA, USA) as manufacturer’s instructions. The plasmid containing the encoded sequence for catalase expression (pcDNA3, Invitrogen, USA) was amplified in competent cells (Invitrogen, Grand Island, USA). Plasmids and Lipofectamine were mixed in Reduced Serum Medium (Invitrogen), and incubated for 20 min at room temperature. For internal control, cells were transfected with empty pcDNA vector.

### Immunoblotting analysis

Cells were lysed by RIPA buffer (50 mmol/L Tris-HCl, pH 7.4; 150 mmol/L NaCl; 1% (v/v) NP-40; 5 mmol/L EDTA; 1 mmol/L DTT; 0.5% sodium deoxycholate; 0.1% SDS; 1 mmol/L Na_3_VO_4_; and 1 mmol/L PMSF) and centrifuged at 12,000×*g* for 30 min at 4°C. Supernatant proteins were determined by Bradford method using BSA as standard and measured at 595 nm. After SDS page separation, proteins were transferred onto a PVDF membrane and revealed by ECL detection reagents. Primary antibodies used in the study included TNF-alpha, TRADD, β-actin, CaM, cytochrome C, calmodulin from Cell signaling technology (Denvers, MA, USA); caspase 8 were from Millipore (Billerica, MA, USA); RIP3 and calpain1 were from Biovision (Milpitas, CA, USA); caspase 3 and were from Abcam (Cambridge, UK).

### Determination of lysosomal membrane permeabilization (LMP)

The aHSCs lysosomal membrane stability was determined by the redistribution of the fluorescent dye, acridine orange [30]. After staining, cells were washed twice with fresh medium at 50 ×*g* for 5 min. to remove excess dye, followed by fluorometrically monitored at Ex495 nm/Em 530 nm by an Olympus IK71 fluorescent microscope (Olympus, Tokyo, Japan). Lysosomal membrane leakiness was determined as cytosolic green fluorescence induced by acridine orange released from lysosomes.

### Calcium assay

The concentration of intracellular Ca^2+^ was measured by calcium assay kit (Cayman, Ann Arbor, MI, USA) as manufacturer’s instructions. The assay is based on the formation of o-cresolphthalein-calcium in alkaline condition, and the produced purple complexes were monitored at absorbance of 575 nm.

### Statistic

The data has been analyzed by Sigma plot version 9.0. Results are presented as mean±SD for individual experiments. Statistical differences (experiment vs. control) were calculated by student's *t*-test and *P*<0.05 was considered as statistically significant.

## Results

### Gallic acid induces oxidative stress leading to significant cytotoxic and antiproliferative effects on aHSCs

**Fig 1:**
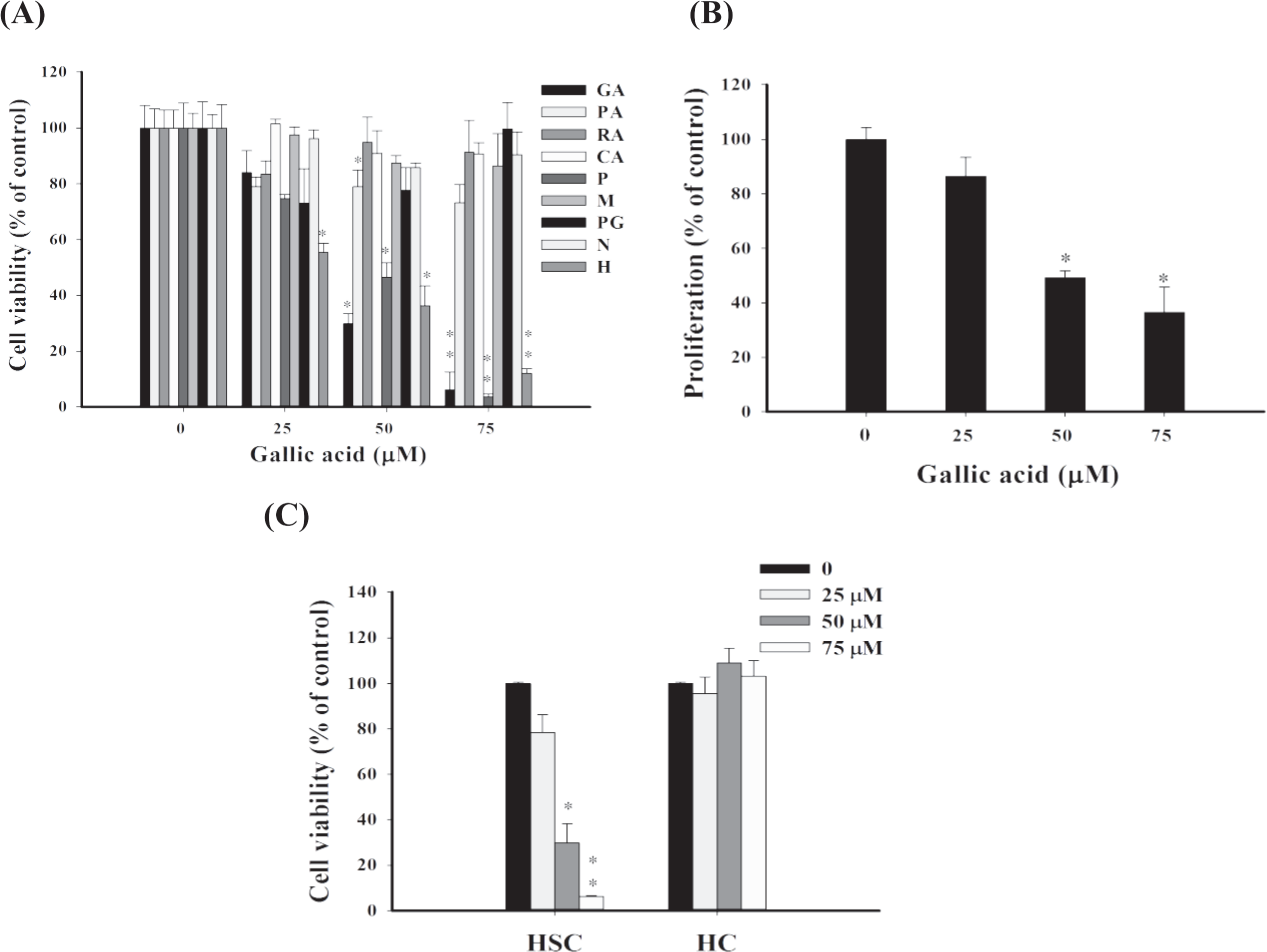
GA induces cytotoxic and anti-proliferative effects on aHSCs. (A) Cell viability was monitored by using an MTT assay. GA analogues were treated with various concentrations (0, 25, 50, 75 μM) for 24 hrs. Gallic acid (GA); 2,3,4-Trihydroxybenzoic acid (TA); Protocatechuic acid (PA); α-Resorcyclic acid (RA); 3,4-Cresotic acid (CA); Methyl-3,4,5-trihydroxybenzoate (M); Propyl gallate (PG); 4-Nitrocatechol (N); 5-Hydroxydopamine hydrochloride (H). (B) Cell proliferation was examined by using a BrdU assay. GA-treated cells demonstrated significant cell proliferative inhibitory effects compared to the control groups. (C) The cytotoxic effects of GA on aHSCs and hepatocytes (HC). Various concentrations of GA (0, 25, 50, and 75 μM) were added to hepatocytes and aHSCs. The cell viability was measured by an MTT assay. Data were expresed as mean±SD from three different experiments. The asterisk (*) indicates a significant difference from control group (* *P*<0.05, **P<0.01).

GA and its analogues with different resonance states that may cause distinct levels of oxidative stress were used to explore GA–induced cytotoxic and antiproliferative effects on aHSCs. The results of the MTT assay indicated that GA and analogues such as pyrogallol (P) and 5-Hydroxydopamine hydrochloride (H) demonstrated dose–dependent cytotoxic effects (25, 50, and 75 μM) on aHSCs after 24 hrs incubation (**Fig. 1A**), with an EC_50_ value of 30.5±1.7, 41.8±1.6, and 35.0±1.0 μM, respectively, while other analogues did not. GA also held significant antiproliferative effects (*P*<0.05) on aHSCs as determined by analyzing the newly synthesized DNA of dividing cells through the BrdU assay. A dose–dependent reduction (55.3±2.3 and 66.9±8.4%) in cell proliferation was observed after 24 hrs incubation at GA concentrations of 50 and 75 μM, respectively (**Fig. 1B**). Notably, in contrast to aHSCs GA showed less cytotoxicity on quiescent HSCs (qHSCs) and no cytotoxicty on normal hepatocytes at GA concentrations of 25, 50, and 75 μM after 24 hrs of incubation (**Fig. 1C**). The cell viability of qHSCs was almost 10 times higher than that of aHSCs (73.4% vs. 7.9%) at GA 75μM after 24 hrs of incubation (**S2 Fig.**).

**Fig 2:**
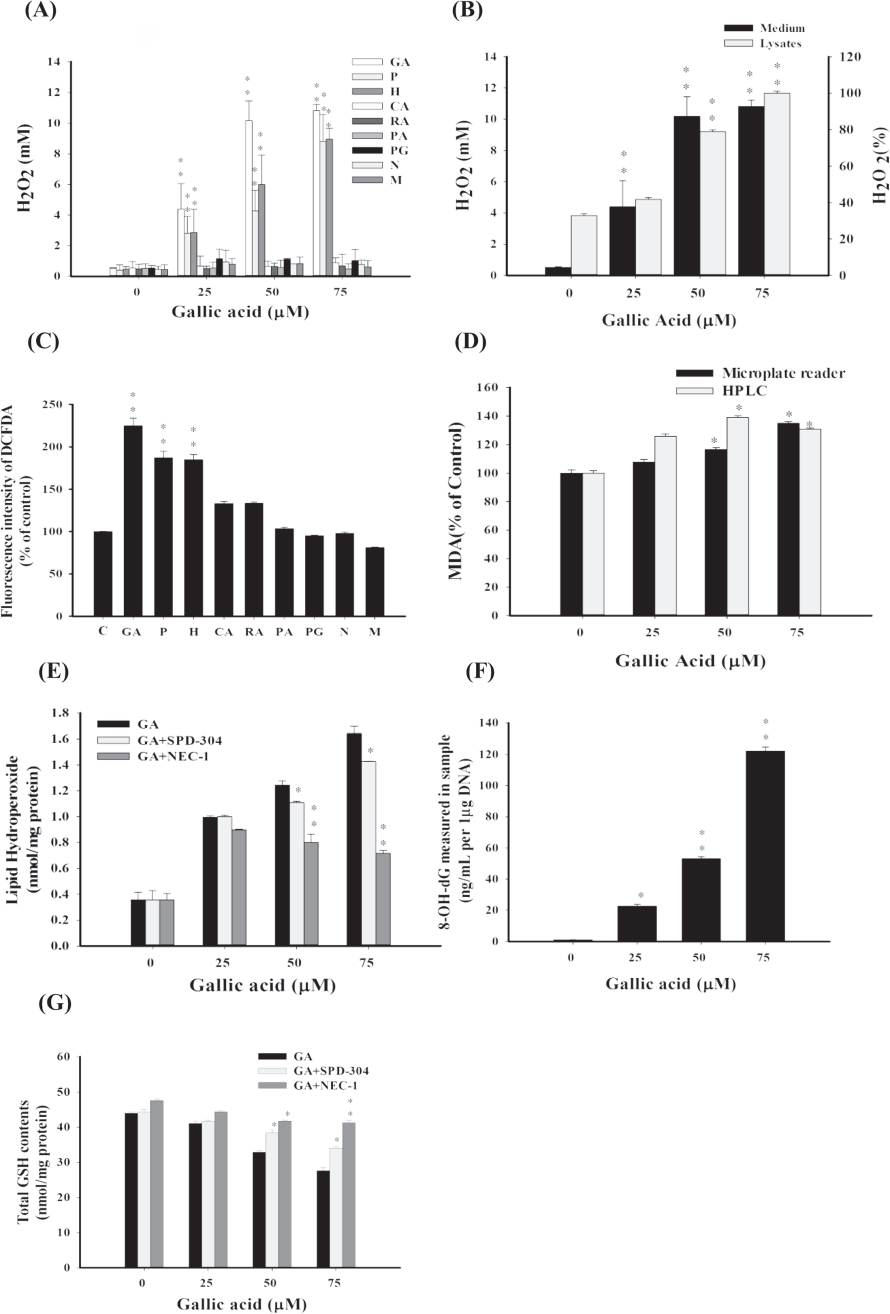
GA induces the formation of H_2_O_2_, ROS, DNA oxidation, and lipid peroxidation in aHSCs. (A) Induction of hydrogen peroxide by GA and GA analogues (0, 25, 50, and 75 μM). (B) GA dose-dependently (0, 25, 50, and 75 μM) increased the production of H_2_O_2_ both in the culture medium and the cytosol. (C) GA-induced ROS, was determined by DCFDA cellular ROS detection assay. GA and analogues GA (50 μM) were treated with aHSCs; the generation of DCF was measured fluorescently. (D) GA-induced lipid peroxidation products, malondialdehyde (MDA), were determined by HPLC or microplate reader after 24 hrs of incubation. (E) Lipid peroxidation products, lipid hydroperoxides, were determined with or without inhibitors of TNF−α and RIP1, SPD-304 (2μM) and Nec-1 (2μg/mL), respectively. (F) Oxidized DNA (8-OH-dG), and (G) total GSH contents were determined. Data were expressed as mean±SD from three different experiments. The asterisk (*) indicates a significant difference from control group (* *P*<0.05, **P<0.01).

Oxidative stress induced by GA and GA analogues was further investigated by analyzing ROS formation. The levels of hydrogen peroxide (H_2_O_2_) in aHSC culture medium was determined after treatment with GA and GA analogues at 25, 50, and 75 μM. Increased levels of H_2_O_2_ were found in the GA, P, and H treated groups (**Fig. 2A**). GA also elevated intracellular content of H_2_O_2_ (**Fig. 2B**). Accumulated intracellular ROS (e.g. hydroxyl and peroxyl radicals) determined by DCFDA cellular ROS detection assay was also observed in the GA, P, and H treated groups (**Fig. 2C**). In addition, GA-induced lipid peroxidation, and oxidative DNA in aHSCs were revealed, as evidenced by dose–dependent formation of MDA (**Fig. 2D**), lipid hydroperoxides (**Fig. 2E**), and 8-oxodG, (**Fig. 2F**), respectively. Intracellular GSH concentration was also decreased with the increase of GA (**Fig. 2G**). These results suggest that GA induces remarkable oxidative stress in aHSCs. Besides, GA analogues like P and H generated significant amount of ROS intracellularly and in culture medium, and induced remarkable cytotoxicity, whereas other analogues induced no or lower levels of ROS and cytotoxicity (**Fig. 1A**). These outcomes might suggest that the involvement of oxidative stress in cell demise was chemical structure specific. Moreover, significant cytotoxicity was observed in aHSCs but not in normal hepatocytes after the treatment of GA, which could be due to the decreased antioxidative activity in aHSCs [19].

### Gallic acid suppresses the catalase activity of aHSC leading to the accumulation of H_2_O_2_

**Fig 3:**
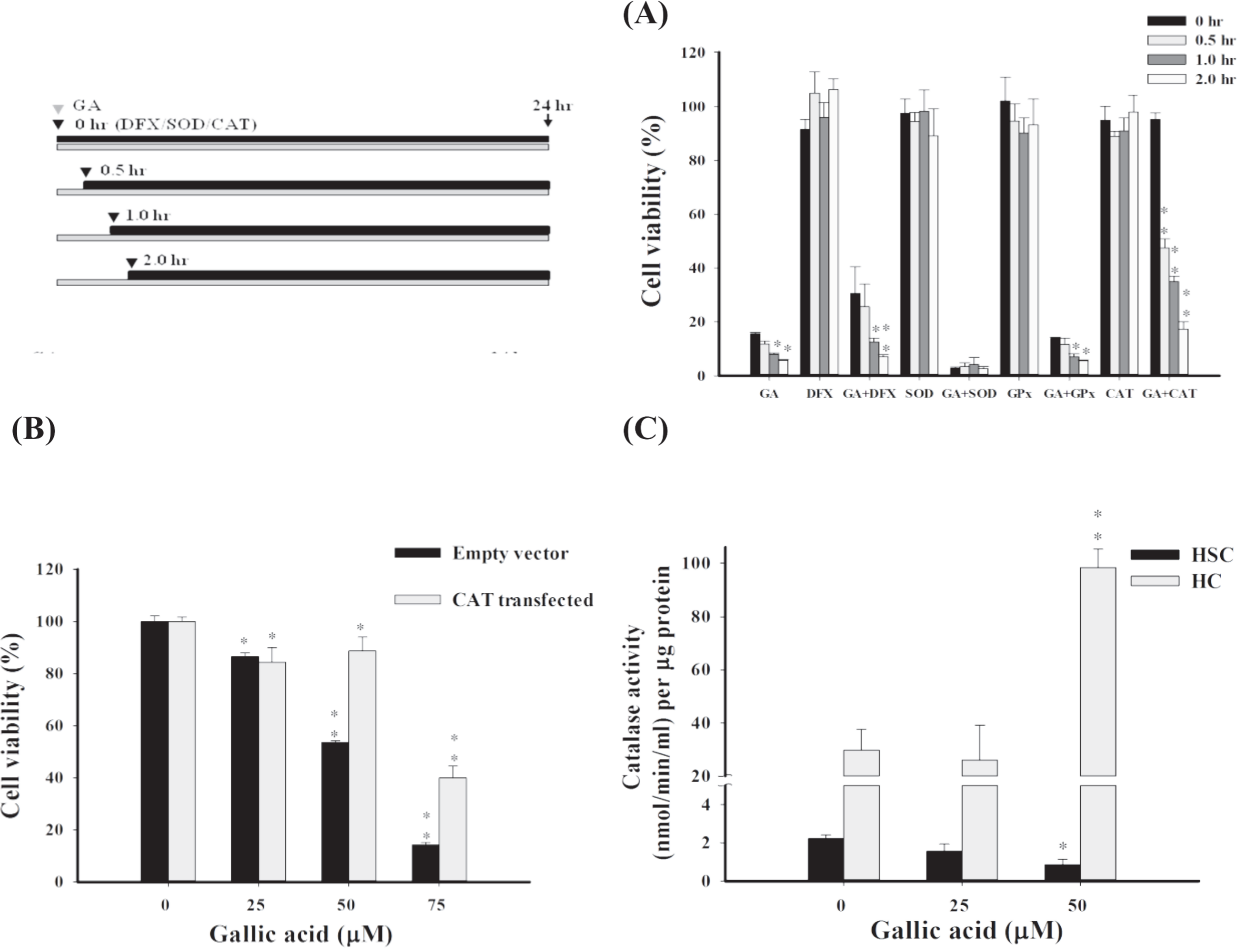
GA down-regulates the activity of catalase in aHSCs. (A) The effect of antioxidants on the aHSC mortality rate. Activated HSCs were pre-treated with 75 μM of GA for 24 hrs, followed by treatements with DFX (100μM), SOD (100 U/mL), and CAT (100 U/mL) at different time intervals (0, 0.5, 1, and 2 hrs) after GA treatment. The cell viability was determined by an MTT assay. (B) The effect of catalase activity on viability in GA treated aHSCs. The aHSCs were transfected with catalase genes and incubated with GA for 24 hrs, followed by measurement of the cell survivability by MTT assay. (C) GA inhibits the activity of catalase in aHSCs but not in hepatocytes. Cells were treated with GA (0, 25, and 50 μM) for 24 hrs before the measurement of catalase activity. * *P*<0.05, **P<0.01.

The accumulation of GA–induced H_2_O_2_ in aHSCs could be resulted from impaired intracellular antioxidant system. To further investigate this assumption, the effect of antioxidant system on cell survivability was then determined (**Fig. 3**). Reagents such as deferoxamine (DFX) (a ferric iron chelator to limit Fenton−like reaction), superoxide dismutase (SOD), and catalase (CAT) were used to reduce oxidative stress. DFX chelates ferric iron to retard Fenton’s reaction and the subsequent radical generation. SOD catalyzes the dismutation of superoxide to oxygen and hydrogen peroxide. Catalase catalyzes the decomposition of hydrogen peroxide to water and oxygen. Activated HSCs were initially incubated with GA, followed by the addition of antioxidants at different time intervals (0, 0.5, 1, and 2 hrs) after GA treatment. After 24 hrs of incubation, the cell viability was determined. **Fig. 3A** indicates that group treated with catalase showed the greatest cell survival promoting effect compared to other antioxidants. Group treated with DFX showed reduced cytotoxic effect in the first two time periods (0 and 0.5 hr) presumably due to the suppression of hydroxyl radical production catalyzed by iron. However, at the late time period (1 and 2 hrs), the cytotoxcity of DFX and GA co−treatment group was similar to that of GA alone, suggesting the critical role of H_2_O_2_ in cytotoxicity. There were significant cytotoxicity and no rescuing effect observed in the groups treated with SOD probably because of the accumulation of H_2_O_2_ resulted by the catalyzation of superoxides. On the other hand, cell survival was significantly promoted in groups treated with catalase, indicating the involvement of H_2_O_2_ in cytotoxicity.

Improved survivability of aHSCs at several levels of GA treatment (25, 50, and 75 μM) was maintained by transducing the catalase genes (**Fig. 3B**). A significant 35.1% and 25.7% recovery (*P*<0.05) at GA concentrations of 50 and 75 μM, respectively, was achieved. Furthermore, the inhibitory potency of GA on the catalase activity was studied. As displayed in **Fig. 3C**, hepatocytes possess higher catalase activity than that of aHSCs under normal conditions. With the addition of GA (25 and 50 μM), the catalase activity of aHSCs was suppressed dose–dependently, whereas the activity of hepatocytes was promoted at higher GA concentrations. These findings suggest that catalase is critical to the survival of aHSCs insulted by GA–induced oxidative stress. It has been reported that restricted catalase activity shows in HSCs once being activated [31]. This could likely make aHSCs more vulnerable to oxidative stress than normal hepatocytes.

### Gallic acid induces TNF−α mediated programmed necrosis in aHSCs

**Fig 4:**
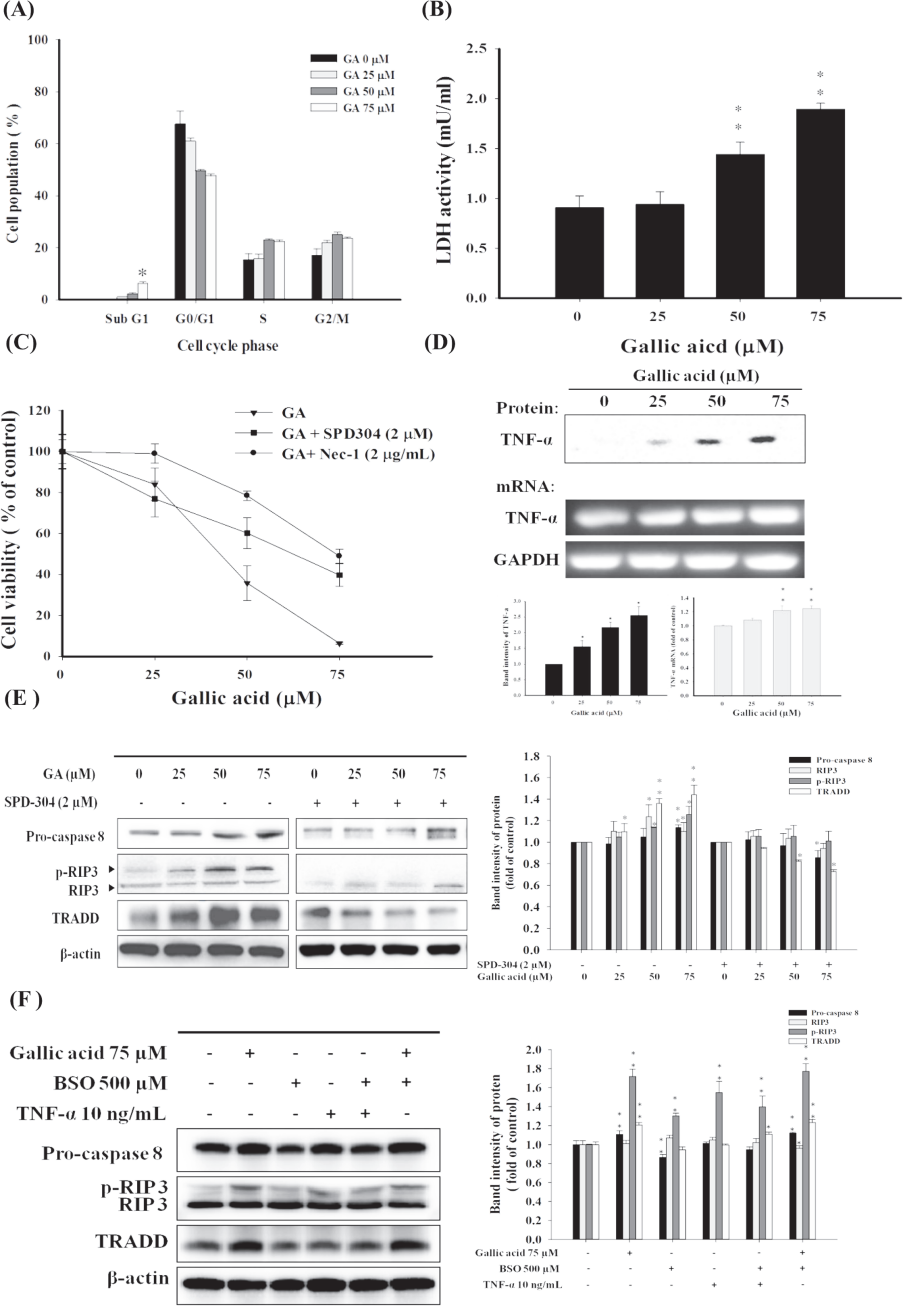
GA induces TNF−α −mediated necroptosis in aHSCs. (A) GA induced low levels of sub-G1 population in aHSCs as analyzed by flow cytometry. (B) Plasma memebrane integrity of aHSCs after GA treatment at designated concentrations was evaulated by LDH assay. (C) Involvement of TNF−α and RIP1 in GA−induced necroptosis. Increased cell viability of aHSCs was obtained through the co−incubation of GA at various concentrations and SPD304 (2μM) or Nec−1 (2μg/mL). (D) GA elicited substantial production of TNF−α as determined by immunoblotting and RT-PCR analysis. Immunoblotting analysis of necroptosis−related factors at various GA concentrations (0, 25, 50, and 75 μM) with or without (E) SPD−304, (F) BSO and TNF−α, co-incubation for 24hrs. Representative immunoblots showed the levels of TRADD, caspase−8, p-RIP3, and RIP3. β−actin was used as an internal control. * *P*<0.05, **P<0.01.

The GA-induced cytotoxic effect on aHSCs was observed in dose-dependent manners (**Fig. 1A**). We then attempted to further reveal the molecular mechanisms by which GA mediated the death of aHSCs. Our cell cycle analysis showed that GA did not provoke significant apoptotic effects on aHSCs (**Fig. 4A, S3 Fig.**). The sub G1 phase showed slight change after GA treatment (25, 50, and 75 μM). However, LDH release (*P*<0.05) appeared with the increase in GA concentrations (25, 50, and 75 μM) (**Fig. 4B**). This dose−dependent LDH release implies the disruption of the plasma membrane and subcellular organelles. Thus, GA might likely mediate a programmed necrotic effect, necroptosis, on aHSCs. It is known that TNF−α pathway has been suggested to be associated with necroptosis, and RIP1 is one of key factors of necroptosis. The TNF−α antagonist, SPD-304, and RIP1 inhibitor, Nec-1, were then used to examine GA-induced programmed necrotic cell death. The addition of SPD-304 and Nec-1 significantly rescued the survivability of aHSCs (**Fig. 4C**), reduced the production of lipid hydroxides (**Fig. 2E**), and increased intracellular GSH (**Fig. 2G**), indicating the involvement of necroptosis in GA-induced programmed cell death.

It is suggested that the activation of RIP3 and TRADD are critical elements of TNF signaling−mediated necroptosis [32]. As shown in **Fig. 4D**, GA induced substantial TNF−α release from aHSCs, which could likely elicit the downstream activation of necroptosis. Additionally, RIP3, the trigger of necroptosis in the TNF−α pathway, along with the up-regulated expression of TRADD and the blocked caspase-8 activity, engages the effector mechanisms of necroptosis [26]. The results of immunoblotting analysis revealed that with the whole lysates of aHSCs, GA significantly up-regulated TRADD and p−RIP3 (1.4 and 1.3−fold, respectively) and down-regulated the activation of caspase-8 (**Fig. 4E)**. The co−treatment of GA (75 μM) and SPD−304 (2 μM), as expected, down−regulated TRADD almost 2−fold (w/o inhibitor vs. w/ inhibitor, 1.41 vs. 0.73) and p−RIP3 1.4−fold (1.32 vs. 0.99) compared to GA alone (**Fig. 4E)**, and promoted caspase 8 activation (1.14 vs. 0.8). These results indicate that GA induced a selective necroptosis in aHSCs by triggering TNF−α signaling pathway.

Based on these findings, GA could likely induce necroptosis partly through the actions of activation of TNF−α pathway, suppression of pro-caspase 8 activation, and depletion of intracellular GSH. Buthionine sulphoximine (BSO), an inhibitor of γ-glutamylcysteine synthetase (γ-GCS) to deplete intracellular GSH, was used in conjunction with TNF−α to investigate whether the factors associated with necroptosis could be provoked. As shown in **Fig. 4F**, BSO alone could significantly elicit the phosphorylation of RIP3 but could not upregulate other factors associated with necroptosis, *e*.*g*. TRADD. On the other hand, the combinatory effects of BSO and TNF−α significantly promoted the activation of RIP3 and TRADD. These results might explain partly the necroptotic mechanisms that GA exerted on aHSCs.

### Gallic acid promotes increased intracellular calcium levels and calpain−1−modulated LMP in aHSCs

**Fig 5:**
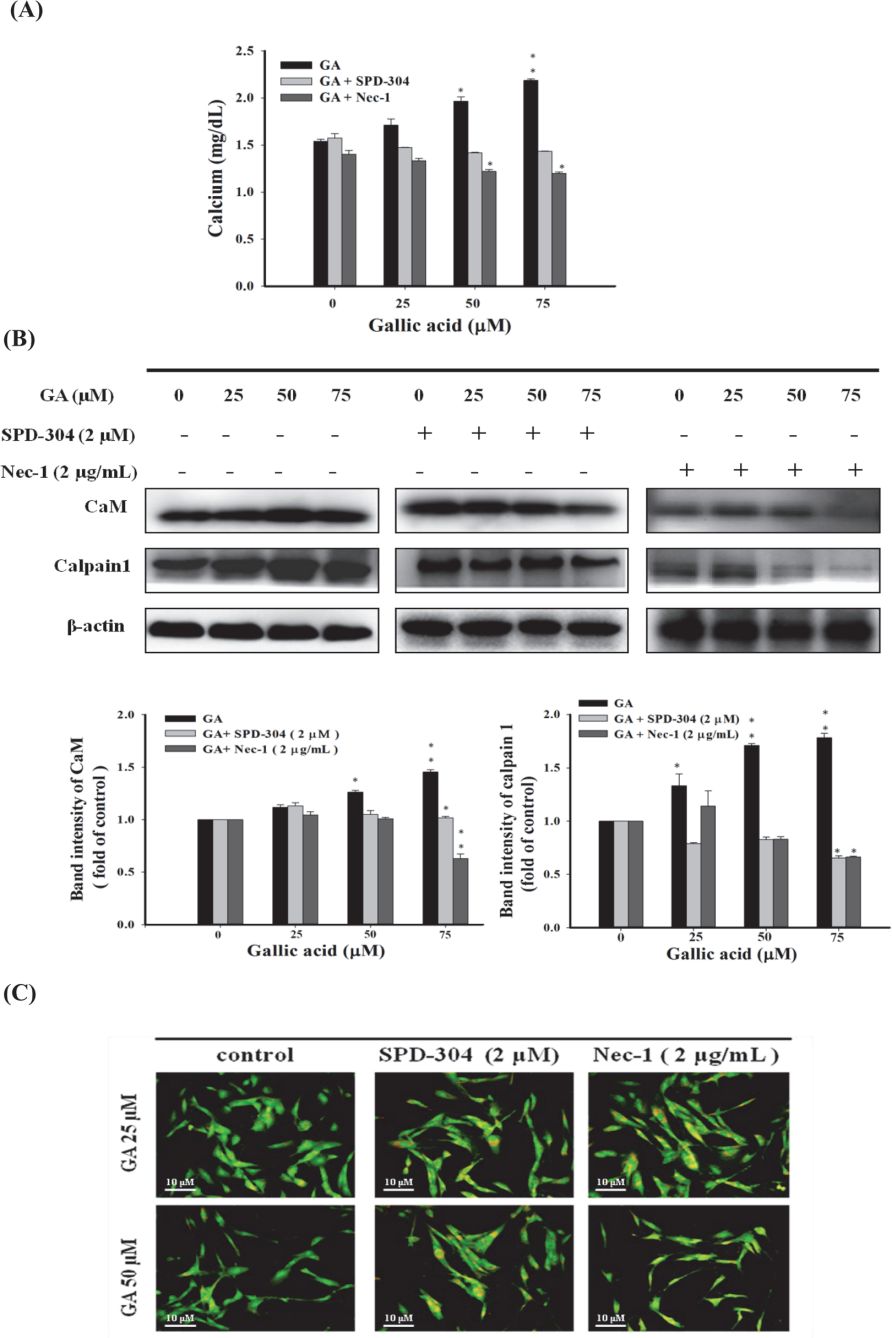
GA−induced necroptosis is associated with Ca^2+^ signaling and lysosomal membrane permeabilization in aHSCs. (A) GA-induced calcium release was regulated by the TNF pathway. The cells were co-treated with GA (0, 25, 50, and 75 μM), and SPD-304 (2 μM) or Nec-1 (2 μg/mL) for 24 hrs, followed by the analysis of cytosolic calcium contents. The results represent the means±SD from three independent experiments. (B) Elevated cytosolic calcium levels triggered by GA upregulated the expression of CaM and calpain 1. The activated HSCs were treated with various concentrations of GA (0, 25, 50, and 75 μM) with or without SPD-304 (2 μM) or Nec-1 (2 μg/mL) for 24 hrs. Representative immunoblots showed the expression of CaM and calpain 1. β-actin was used as an internal control. * *P*<0.05, **P<0.01. (C) GA induces lysosomal membrane permeabilization in aHSCs. The effects of GA on lysosomal stability. The activated HSCs were treated with GA (25 and 50 μM) and with or without SPD-304 (2 μM) or Nec-1 (2 μg/mL) for 24 hrs. The activated HSCs were stained with aridine orange to determine the integrity of the lysosomes. (Scale bars, 10μm).

**Fig. 5A** indicated that the intracellular calcium level rose with the elevation of GA concentrations (25, 50, and 75 μM) (*P*<0.05). The accumulation of calcium was suppressed by co−treatment with GA (75 μM) and SPD−304 (GA alone vs. GA/SPD−304, 2.19 vs. 1.44 mg/dL, respectively). The addition of Nec−1 also suppressed GA−induced Ca^2+^ elevation (GA alone vs. GA/Nec-1, 2.19 vs. 1.25 mg/dL, respectively, at GA 75 μM). These results indicate that GA−induced Ca^2+^ accumulation was through death receptor (DR)−elicited signaling. Molecules that associated with calcium-modulated necroptosis such as intracellular calcium concentration regulator, calmodulin (CaM), and calcium-activated neutral protease, calpain 1, were then examined. The active form of calpain executes lysosomal membrane permeabilization (LMP), which causes lysosome rupture and the spillage of acidic lysosomal contents to mediate cytoplasm acidification and degradation [33]. The results of the immunoblotting analysis indicate that GA remarkably up−regulated the expression of CaM and calpain 1, but the elevation was suppressed by the treatment of SPD−304 and Nec−1 **(Fig. 5B),** suggesting that GA triggers the process of necroptosis through the modulation of calcium signaling.

Next, calpain−induced lysosomal membrane permeabilization (LMP) during GA−induced necroptosis was investigated by lysosomal staining with acridine orange. As shown in **Fig. 5C**, low level of orange fluorescence was observed in cells treated with GA alone, whereas increased orange fluorescence appeared upon the addition of SPD−304 and Nec−1, indicating the presence of intact acid organelle such as lysosome, after the treatment of inhibitors. These results indicate that either blocking TNF−α signalling or RIP1 remarkably arrested the process of GA-induced LMP, which rescued the subsequent cell viability (**Fig. 4B**). Collectively, our data demonstrated that GA−induced TNF−α −mediated necroptosis in aHSCs was elicited by triggering RIP1 and RIP3 necroptosome, followed by the modulation of Ca^2+^ signaling to execute LMP through calpain1 activation.

### Inhibition of RIP1 activities diverts GA−induced necroptosis to apoptosis

**Fig 6:**
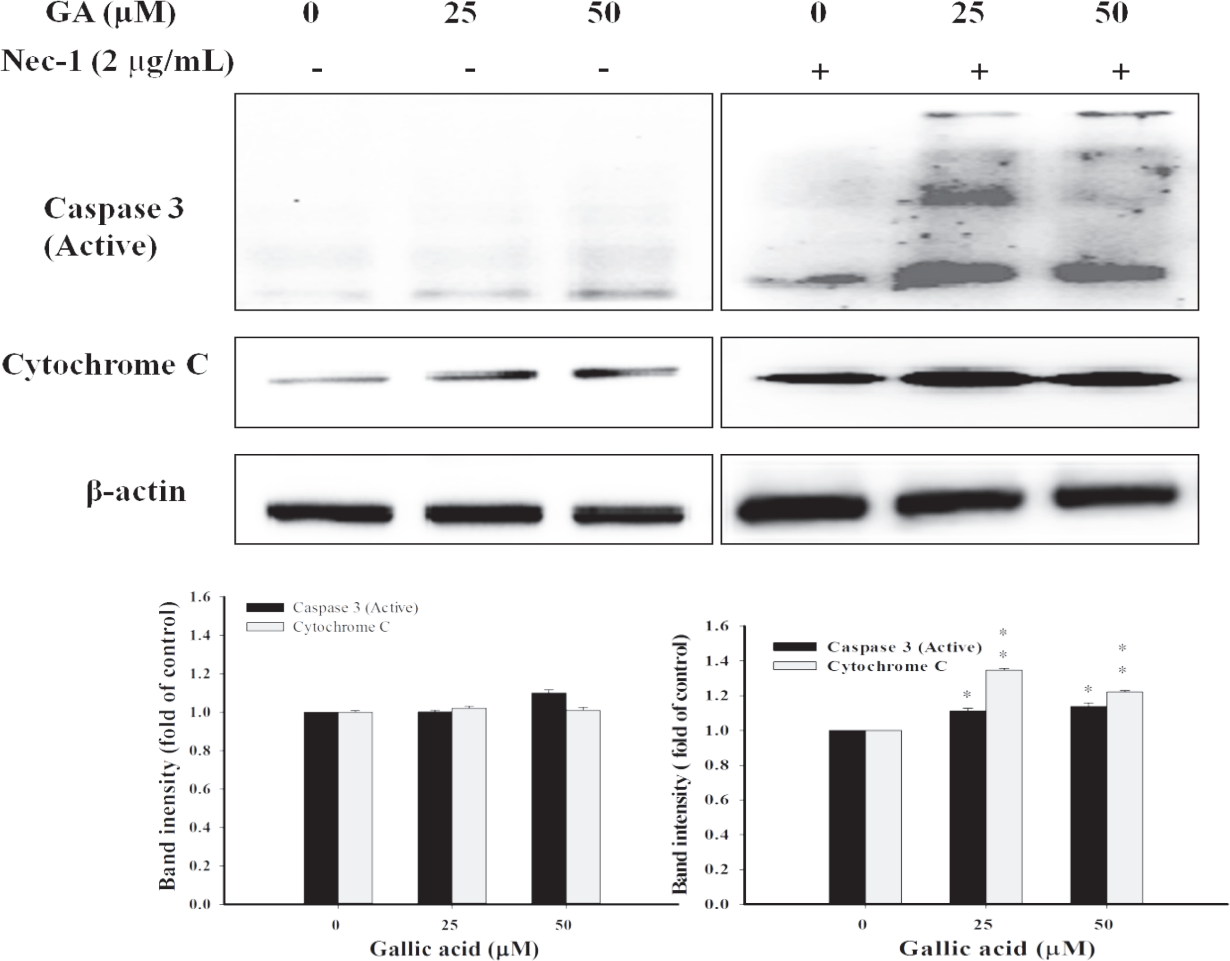
Inhibition of RIP1 diverts GA-induced necroptosis to apoptosis. Dose-dependent activation of caspase-3 and cytochrome c in aHSCs co-treated with GA and Nec-1. The cells were treated with GA (0, 25, and 50 μM) for 24 hrs in the presence of Nec-1 (2 μg/mL), followed by the analysis of apoptosis-related protein expression by immunoblotting. Representative immunoblots showed the levels of active caspase-3 and cytochrome c. β-actin was used as an internal control. * *P*<0.05, **P<0.01.

It has been reported that necroptosis is reciprocal to apoptosis when the apoptotic signaling is blocked [34]. Therefore, we attempted to study whether blocking the GA-induced signals of necroptosis could divert cell death toward apoptosis. Various concentrations of GA were concurrently added with Nec−1 to aHSCs. **Fig. 6** reveals that under apoptosis−competent condition (with Nec−1), GA (25 and 50 μM) significantly activated caspase 3 and released cytochrome c to the cytoplasm. On the other hand, under apoptosis−deficient condition (without Nec−1), no significant activation of caspase 3 and cytochrome c was observed. Based on these results, the activity of RIP1 was required in GA−triggered aHSC necroptosis. In addition, the diversion of GA−triggered necroptosis to apoptosis verified the reciprocal relationship of these two cell death processes to ensure cell termination under stimuli conditions [34].

## Discussion

In the present study, we aimed to investigate the molecular mechanisms of programmed cell death that GA exerted in active hepatic stellate cells, a key factor associated with hepatic fibrosis. We revealed that GA promoted necroptotic cell death through the induction of TNF–α–mediated necroptosis. GA induced significant oxidative stress as observed by the depletion of intracellular GSH, the formation of intracellular aldehyde (e.g., malondialdehyde, MDA) and hydrogen peroxide, as well as ROS accumulation, which led to subsequent cytotoxicity. It is intriguing that the GA esters, methyl 3,4,5,-trihydroxy-benzoate (M) (–COOCH_3_ at C_1_) and propyl 3,4,5-trihydroxy-benzoate (PG) (–COO(CH_2_)_2_CH_3_ at C_1_), showed much lower levels of ROS formation and cytotoxicity than those of GA (–COOH at C_1_), pyrogallol (P) (–H_2_ at C_1_), and 5-Hydroxydopamine hydrochloride (H) (–C_2_H_4_NH_2_ at C_1_). Presumably GA, P, and H are in more resonance forms than M and PG leading to higher levels of ROS formation and cytotoxicity resulted.

GA-induced oxidative damage and cytotoxic effects were low in hepatic cells but were high in aHSCs, which could be attributed to the activity of antioxidative systems, such as catalase, a critical regulator of intracellular ROS levels. Hepatocytes hold potent catalase activity and can eliminate GA−induced oxidative stress displaying enhanced cell survivability. Suppressed catalase activity has been addressed in hepatoma cells and activated HSCs [31,35]. Mechanisms involved in decreasing catalase activity have been reported in hepatoma cells due to the genomic methylation of CpG sites in the catalase promoter [21,35], which might also apply to aHSCs during transformation.

**Fig 7:**
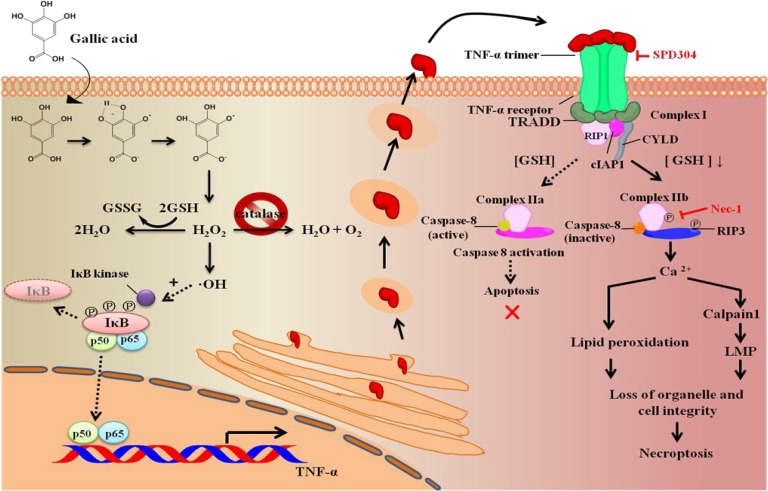
Schematic illustration of the signaling pathway of GA-induced necroptosis. GA induced the bioactivities of aHSCs in several ways: GA 1) depletes GSH and 2) suppresses catalase activity, and 3) promoted the expression of TNF−α and the formation of TNFR1-elicited necrosome complexes, resulting in the accumulation of Ca^2+^ and activation of calpain 1 to attack the lysosomes, leading to necroptosis.

Our results indicated that GA significantly promoted the secretion of TNF–α and the production of RIP1, reduced intracellular GSH levels, and inhibited the activation of caspase–8 in aHSCs. These observations may suggest the involvement of necroptosis. Moreover, GA also induced several cellular events such as intracellular Ca^2+^ influx, lipid peroxidation, and lysosomal disruption (LMP) by Ca^2+^ influx activated calpains [36], which are all typical characteristics of necroptosis. Inactive form of caspase–8 integrated with RIP3 leads to the subsequent mobilization of calpain and the promotion of LMP, causing the loss of organelle and cell integrity, and finally leading to necroptosis. These phenomena summarized in **Fig. 7** indicate the processing of necroptosis in GA treated aHSCs.

The reduction of intracellular GSH levels caused by GA–induced oxidative stress could be essential to the diversion of programmed cell death from apoptosis, which is reportedly occurred in several GA–induced cell deaths, to necroptosis. Reduced levels of GSH have been seen to repress the undergoing of apoptosis. Direct depletion of GSH under pro-oxidative condition has been indicated to prevent CD95—and TNFR1–mediated hepatocyte apoptosis *in vivo* [37]. The oxidized GSH, GSSG, is also shown to blockade apoptosome–mediated caspase–3 activation [37]. Further, the activation of caspase such as caspase–8 is suggested under a reducing environment. This caspase requires antioxidants at death–inducing signaling complex (DISC) for activation [37]. Therefore, the accumulation of intracellular hydrogen peroxide and the depletion of GSH induced by GA could likely impair the activation of caspase–3 and 8, leading to necroptosis in aHSCs.

The reciprocal backup relationship of apoptosis and necroptosis has been addressed [34] to ensure cell termination under stimuli conditions. Inactivation of RIP1 by Nec–1 diverts GA–triggered necroptosis to apoptosis as evidenced by the increased level of cytocrome c and the activation of caspase–3. RIP1 plays several roles in the promotion of necroptosis. RIP1 is not only an element of necrosome, but also a mediator in phosphorylating an anti-apoptotic factor, STAT3, at Ser727, which enables the activated molecule to interact with GRIM-19 resulting in the subsequent translocation to mitochondria [38]. This leads to an apoptosis–deficient situation, and provokes TNF–induced necroptosis. RIP1 has been seen to mediate caspase inhibitor–induced TNF–α production [39], and TNF–induced ROS generation [40] to regulate the progression of necroptosis. Thus, the inhibition of RIP1 by Nec–1 would restrict the undergoing of necroptosis. On the other hand, Nec–1, not an antioxidant, is reported to be able to resume intracellular reducing environment due to the suppressive ability in GSH depletion and ROS formation [41]. Accordingly, in addition to be a RIP1 inhibitor, Nec–1 may exercise its “antioxidative” character to halt necroptosis, which usually dysregulate cellular redox metabolome through the depletion of NAD^+^, NADPH, and GSH [42].

## Conclusion

GA-induced apoptosis has been reported elsewhere, however, GA elicits necroptosis in aHSCs was first reported herein. GA elicits TNF signaling pathway that promotes necroptosis in aHSCs. The oxidative stress induced by GA may trigger the production of TNF–α, which evokes the downstream signaling of necroptosis, including the formation of necrosome (activation of RIP1, RIP3, and inactivation of caspase–8) and the subsequent events such as intracellular Ca^2+^ influx, lipid peroxidation, and lysosomal disruption (LMP) by Ca^2+^ influx activated calpains. This is the first report that indicates GA–induced necroptosis in aHSCs, which may provide an alternative strategy for the amelioration of liver fibrosis, in addition to the anti–oxidative activity of this phenolic compound. The intermittent molecules of TNF–α signaling pathway responsible for TNF–α–mediated necroptosis have not yet been clearly asserted. The examination of GA–induced cell death signals that propagate further will be focused on the investigation of cellular redox metabolome signaling associated with other modes of regulated necrosis.

## Supporting Information

S1 Fig Molecular structures of GA and its analogues. 3,4,5-trihydroxybenzoic acid (gallic acid, GA), 3,4-Diihydroxybenzoic acid (Protocatechuic acid, PA), 3,5-Diihydroxybenzoic aicd (α -resorcyclic acid, RA), 3-Hydroxy-4-methylbenoic acid (3,4-cresotic acid, CA), Pyrogallol (P), Methyl 3,4,5,-trihydroxy-benzoate (M), Propyl 3,4,5-trihydroxy-benzoate (PG), 4-Nitrocatechol (N), 5-Hydroxydopamine hydrochloride (H).
(TIF)

S2 Fig The cytotoxic effects of GA on qHSCs. Various concentrations of GA (0, 25, 50, and 75 μM) were added to qHSCs. The cell viability was measured by an MTT assay. Data were expressed as mean±SD from three different experiments. The asterisk (*) indicates a significant difference from control group (* *P*<0.05, **P<0.01).
(TIF)

S3 Fig GA induces low levels of sub-G1 population in aHSCs. Activated HSCs were cultured in serum free medium with GA (0, 25, 50, 75 μM) for 24 h, followed by flow cytometric analysis with ethanol (70%) fixation and propidium iodide (PI) labeling.
(TIF)
