# Supplementary figures and images for: Gallic Acid Induces Necroptosis via TNF–α Signaling Pathway in Activated Hepatic Stellate Cells
Source: PLoS One. 2015 Mar 27;10(3):e0120713. doi: 10.1371/journal.pone.0120713 (PMC4376672; doi:10.1371/journal.pone.0120713)

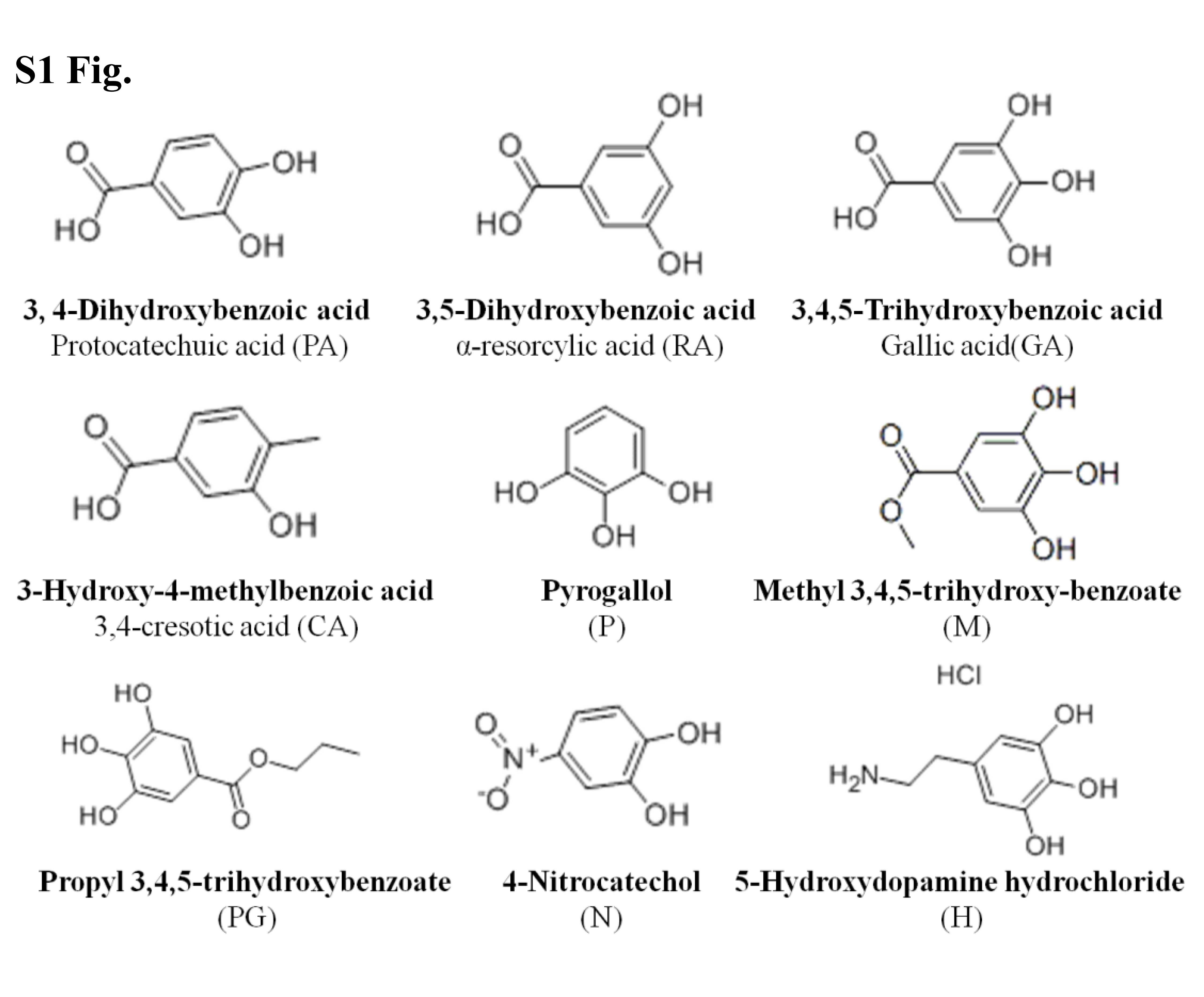

Supplement: S1 Fig — 3,4,5-trihydroxybenzoic acid (gallic acid, GA), 3,4-Diihydroxybenzoic acid (Protocatechuic acid, PA), 3,5-Diihydroxybenzoic aicd (α -resorcyclic acid, RA), 3-Hydroxy-4-methylbenoic acid (3,4-cresotic acid, CA), Pyrogallol (P), Methyl 3,4,5,-trihydroxy-benzoate (M), Propyl 3,4,5-trihydroxy-benzoate (PG), 4-Nitrocatechol (N), 5-Hydroxydopamine hydrochloride (H). (TIF) [file pone.0120713.s001.tif]

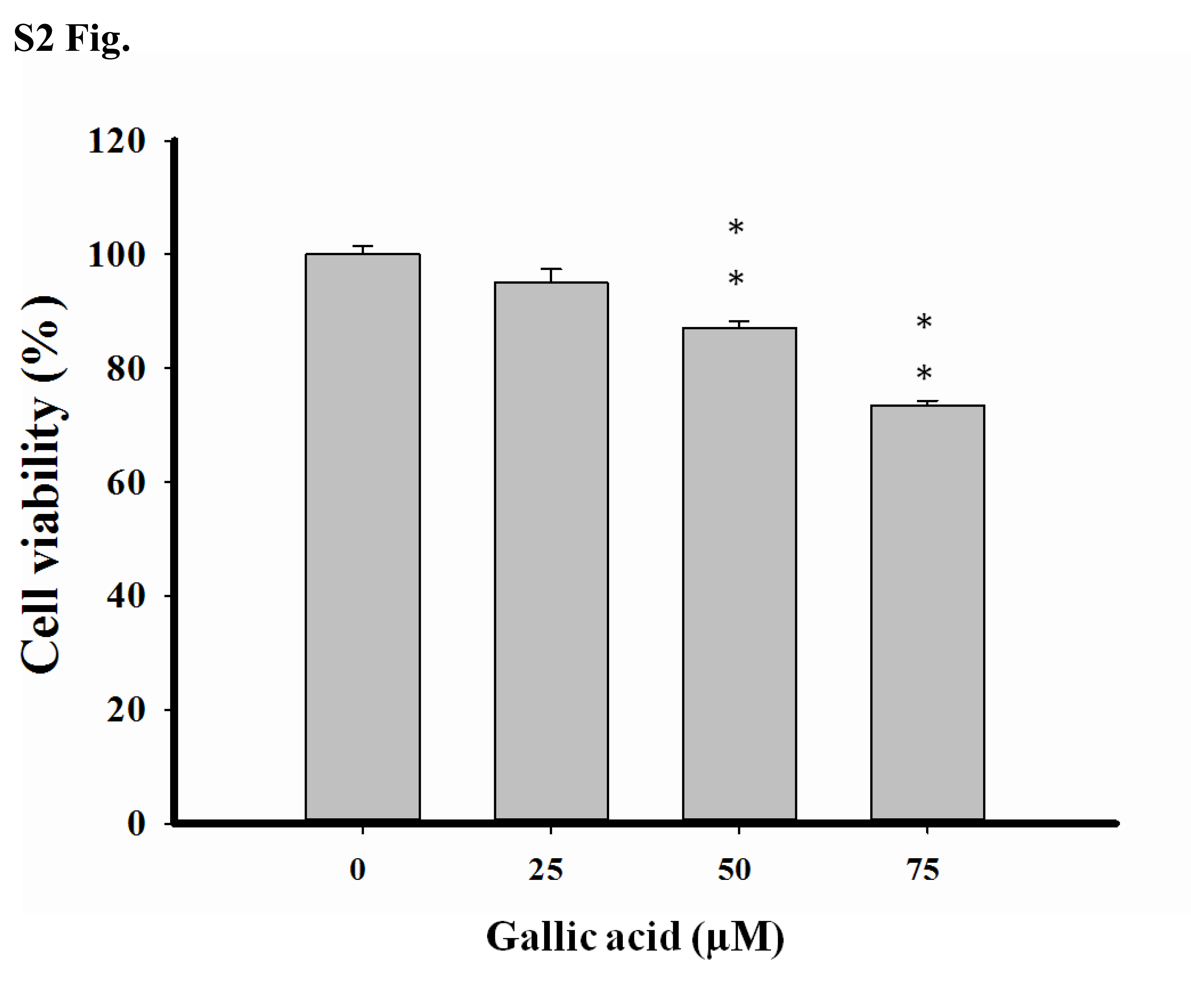

Supplement: S2 Fig — Various concentrations of GA (0, 25, 50, and 75 μM) were added to qHSCs. The cell viability was measured by an MTT assay. Data were expressed as mean±SD from three different experiments. The asterisk (*) indicates a significant difference from control group (* P<0.05, **P<0.01). (TIF) [file pone.0120713.s002.tif]

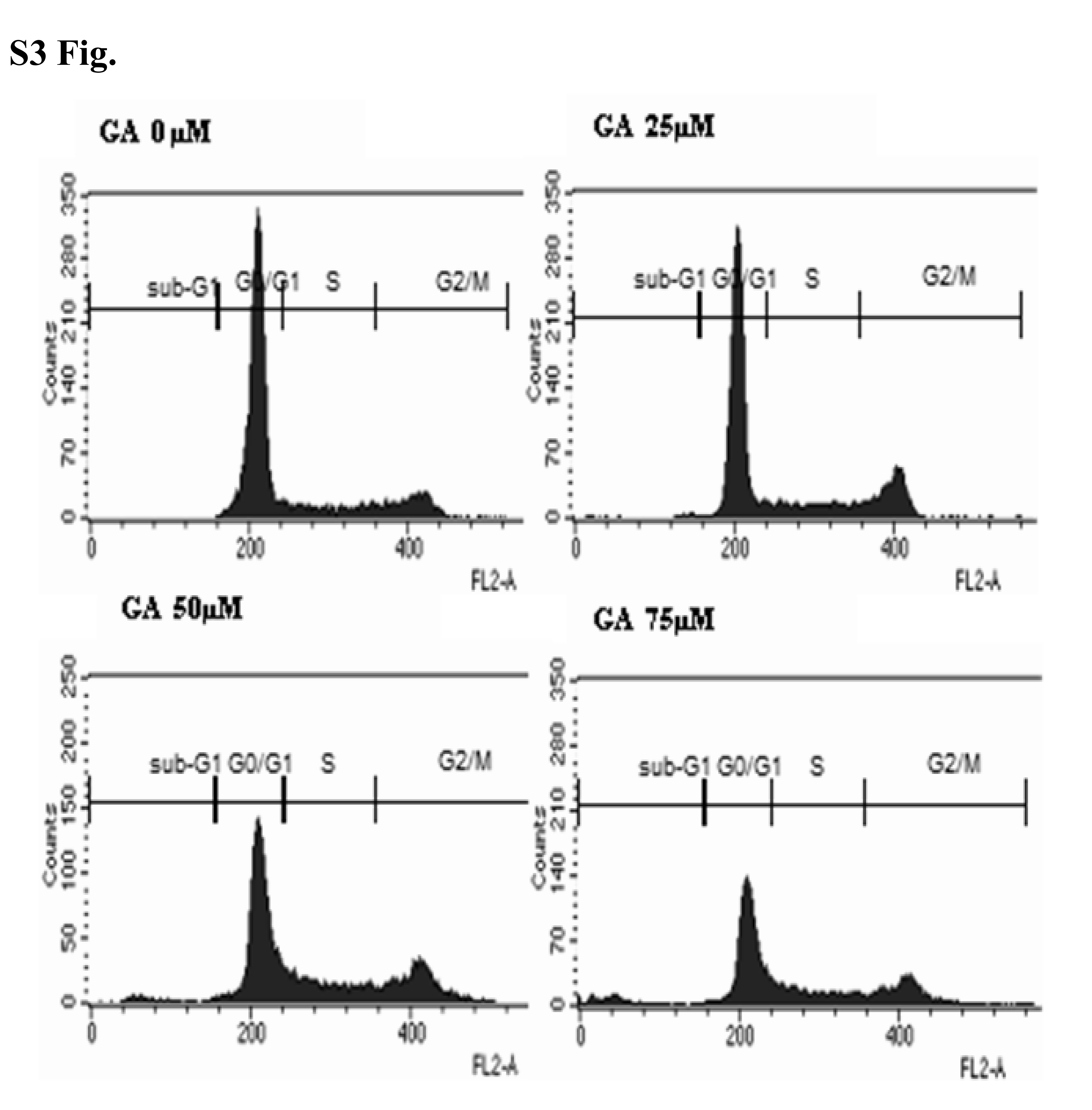

Supplement: S3 Fig — Activated HSCs were cultured in serum free medium with GA (0, 25, 50, 75 μM) for 24 h, followed by flow cytometric analysis with ethanol (70%) fixation and propidium iodide (PI) labeling. (TIF) [file pone.0120713.s003.tif]
